# A small RNA controls bacterial sensitivity to gentamicin during iron starvation

**DOI:** 10.1371/journal.pgen.1008078

**Published:** 2019-04-22

**Authors:** Sylvia Chareyre, Frédéric Barras, Pierre Mandin

**Affiliations:** 1 Aix Marseille Univ–CNRS, UMR 7243, Laboratoire de Chimie Bactérienne, Institut de Microbiologie de la Méditerranée, Marseille, France; 2 Institut Pasteur, Département de Microbiologie, SAMe Unit, ERL CNRS, Paris, France; Universidad de Sevilla, SPAIN

## Abstract

Phenotypic resistance describes a bacterial population that becomes transiently resistant to an antibiotic without requiring a genetic change. We here investigated the role of the small regulatory RNA (sRNA) RyhB, a key contributor to iron homeostasis, in the phenotypic resistance of *Escherichia coli* to various classes of antibiotics. We found that RyhB induces phenotypic resistance to gentamicin, an aminoglycoside that targets the ribosome, when iron is scarce. RyhB induced resistance is due to the inhibition of respiratory complexes Nuo and Sdh activities. These complexes, which contain numerous Fe-S clusters, are crucial for generating a proton motive force (pmf) that allows gentamicin uptake. RyhB regulates negatively the expression of *nuo* and *sdh*, presumably by binding to their mRNAs and, as a consequence, inhibiting their translation. We further show that Isc Fe-S biogenesis machinery is essential for the maturation of Nuo. As RyhB also limits levels of the Isc machinery, we propose that RyhB may also indirectly impact the maturation of Nuo and Sdh. Notably, our study shows that respiratory complexes activity levels are predictive of the bacterial sensitivity to gentamicin. Altogether, these results unveil a new role for RyhB in the adaptation to antibiotic stress, an unprecedented consequence of its role in iron starvation stress response.

## Author summary

Understanding the mechanisms at work behind bacterial antibiotic resistance has become a major health issue in the face of the antibiotics crisis. Here, we show that RyhB, a bacterial small regulatory RNA, decreases the sensitivity of *Escherichia coli* to the antibiotic gentamicin when iron is scarce, an environmental situation prevalent during host-pathogen interactions. This phenotypic resistance is related to the activity of the respiratory complexes Nuo and Sdh, which are producing the proton motive force allowing antibiotic uptake. Altogether, this study points out to a major role for RyhB in escaping antibacterial action.

## Introduction

The emergence and spread of bacterial multi-resistance to antibiotics has become a major health issue in the last decades, urging for the development of new anti-bacterial molecules and for a better understanding of the molecular mechanisms at work behind bacterial resistance [1,2]. While acquired resistance mechanisms (acquisition of genes or mutations that confer resistance) have long been the main focus of attention, less is known about “phenotypic” resistance, which is the process in which a bacterial population becomes transiently resistant to an antibiotic without requiring a genetic change [3–5]. For instance, this kind of resistance has been associated with specific processes such as stationary growth phase, persistence and metabolic changes, reinforcing the idea that the environment encountered by the pathogen is a key determinant for antibiotic susceptibility [6].

Change in utilization of iron-sulfur (Fe-S) cluster biogenesis machineries in *Escherichia coli* gives a striking example of phenotypic resistance [7]. Fe-S clusters are ubiquitous and ancient cofactors used in a plethora of biological processes, such as metabolism and respiration [8,9]. In *E*. *coli*, Fe–S clusters are formed and brought to target proteins thanks to two dedicated biogenesis systems: the so called “housekeeping” Isc machinery, which homologs are found in mitochondria of eukaryotic organisms, and the stress-responsive Suf system, in which homologs are found in chloroplasts of plants [10,11]. These systems are responsible for the maturation of more than 150 Fe-S cluster containing proteins in *E*. *coli*, notably numerous proteins contained in the main respiratory complexes I (Nuo) and II (Sdh) [12–14]. Strikingly, it was shown that impairment of the *E*. *coli* Isc machinery enhances resistance to aminoglycosides, a well-known class of antibiotics that target the ribosome [7]. This resistance is due to a deficiency in the maturation of the respiratory complexes in *isc* mutants, which in turn leads to a decrease in the proton motive force (pmf) that is essential for aminoglycosides uptake [15]. Consistently, the Suf machinery was shown to maturate inefficiently the Fe-S cluster containing proteins of the respiratory complexes, although the molecular reason for this still remains unclear [7]. Overall this study predicted that an environmental signal that induces the switch from Isc to Suf should induce a transient resistance to aminoglycosides.

Iron starvation is one such signal as it decreases the expression of the *isc* operon and enhances that of the *suf* operon. The small RNA RyhB was proposed to participate to this transition [16]. RyhB is one of the most studied sRNAs to date in *E*. *coli* [17–19]. RyhB is regulated by Fur, the main regulator of Fe-homeostasis in many bacteria and is expressed during iron starvation [20,21]. When iron becomes limiting in the medium, RyhB base-pairs and represses the translation of more than 100 mRNA targets that encode non-essential iron-utilizing proteins, thus engaging an “iron sparing” response and redirecting iron consumption in the cell [19]. Notably, RyhB was shown to base-pair to the *iscRSUA* mRNA [16]. RyhB induces the degradation of the 3’ part of the mRNA that contains *iscSUA*, in this way limiting Isc levels, while the 5’ part that encodes *iscR* remains stable. While RyhB repression of *isc* expression is rather modest, it may play a more important role by indirectly contributing to the Isc to Suf transition. Indeed, IscR is the major regulator of Fe-S clusters homeostasis and is itself a Fe-S cluster protein maturated by Isc [22]. Likewise inhibition of Isc functioning is predicted to yield to accumulation of IscR in its apo-form, which actually acts as an activator of the *suf* operon [23]. Note that the *suf* operon is also under Fur repression, which is alleviated under iron limitation.

Iron homeostasis has been shown to modify the sensitivity of bacteria to a number of antibiotics, although the molecular basis behind this is not always clear [24]. Here we asked if the sRNA RyhB could participate in phenotypic resistance to various antibiotics during iron starvation. We found that RyhB is necessary to induce gentamicin phenotypic resistance in low iron conditions. By further investigating the mechanism by which RyhB controls this phenotypic resistance, we show that RyhB controls entry of aminoglycosides in the cell by inhibiting the activity of the two pmf-producing respiratory complexes Nuo and Sdh.

## Results

### RyhB is involved in sensitivity to the aminoglycoside gentamicin

We first investigated whether RyhB has any role in resistance against different classes of antibiotics during iron starvation. To mimic iron starvation, we treated the medium with 250 μM of dipyridyl (DIP), a strong iron chelator. We chose this concentration of DIP because it is known to induce RyhB synthesis [25,26]. Growth of both the WT and *ryhB* mutant strains were slightly affected by depleting iron from the medium, but importantly, doubling time of the *ryhB* mutant was identical to that of the WT strain (S1 Fig). This observation precludes any difference in antibiotic sensitivity between strains to be attributed to difference in growth properties. We then performed antibiotic killing assays by growing wild type (WT) and *ryhB* mutant cells in LB medium added or not with DIP. Antibiotics were added when cells reached early exponential phase (OD_600_ = 0.2) and the number of survivors was determined by counting the number of colony forming units (c.f.u) after 3 hours of incubation. Four different major classes of antibiotics were tested: aminoglycosides (gentamicin), β-lactams (ampicillin), fluoroquinolones (norfloxacin), and tetracycline.

**Fig 1:**
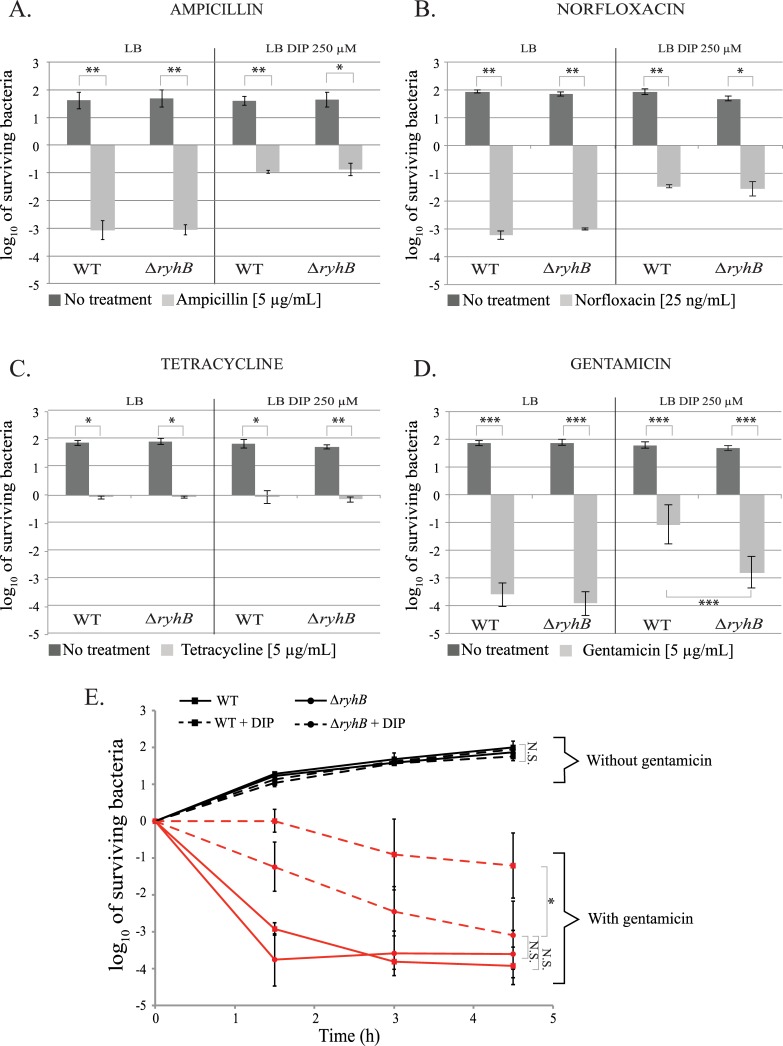
RyhB is involved in gentamicin resistance during iron starvation. A to D: strains were grown in LB (left panels) or in LB with DIP (250 μM) (right panels) for 3 h with or without the following antibiotics A: ampicillin (5 μg/mL); B: norfloxacin (25 ng / mL); C: tetracycline (5 μg/mL) and D: gentamicin (5 μg/mL). Colony forming units were counted to determine the number of surviving bacteria. Points were normalized relatively to t0 and plotted as log_10_ of surviving bacteria. The absolute c.f.u. at time-point zero was ≈ 5.10^7^ c.f.u. / mL for each sample. Error bars represent the standard deviations of three independent experiments. Statistical analyses were performed with Student’s T-test: *p < 0.05; **p < 0.01; ***p < 0.001. E: WT (squares) and *ryhB* mutant (circles) strains were grown in LB (regular lines) or LB depleted for iron (dashed lines) with (red curves) or without (black curves) gentamicin. The number of c.f.u. was determined at different times. Error bars represent the standard deviations of three independent experiments. Statistical analyses were performed with Student’s T—test: *p < 0,05; N.S.: Not significant.

Iron chelation did not protect cells against tetracycline (Fig 1C). In contrast, adding DIP to the medium protected the WT and *ryhB* mutant strains against toxicity of ampicillin, norfloxacin and gentamicin (Fig 1A and 1B). The protective effect of iron deprivation for these antibiotics has already been observed and its underlying cause has been greatly debated [7,24,27,28]. As cells were protected independently of *ryhB*, we did not pursue these antibiotics further. In contrast, WT cells were protected against gentamicin when DIP was added to the medium, but this protection effect was lost when cells were mutated for *ryhB*. (Fig 1D). This result thus suggested that RyhB is involved in the protection of bacterial cells against aminoglycosides during iron starvation.

Next, we performed gentamicin kinetic killing assays by counting the number of WT or *ryhB* survivors at different time intervals after adding gentamicin. In this experiment, both the WT and *ΔryhB* strains showed the same profile when grown in LB (Fig 1E). In both cases, the majority of the cells were rapidly killed after 1 h 30 min of incubation with gentamicin (5 logs of killing). Again, addition of DIP to the medium had a ≈ 4 log protective effect against gentamicin on WT cells as early as 1 h 30 min post addition of the antibiotic. Cells then remained mainly resistant to gentamicin during the course of the experiment. In contrast, the *ryhB* mutant gradually became as sensitive as cells grown in the absence of DIP (see 4 h 30 min time point), although killing kinetics were slightly slower than in presence of iron.

Finally, effect of RyhB on gentamicin efficacy during iron starvation was estimated by defining minimum inhibitory concentration (MIC) values for both WT and *ryhB* mutants in presence of increasing concentration of gentamicin, with or without DIP. MIC value for the WT strain almost doubled when the cells were grown in the presence of DIP (S2 Fig). In sharp contrast, MIC values of the *ryhB* mutant were the same in the presence or absence of DIP. Altogether, these results indicated that RyhB is needed for the phenotypic resistance of *E*. *coli* to gentamicin in low iron condition.

### RyhB decreased sensitivity to gentamicin is dependent on Nuo and Sdh

Entry of aminoglycosides is dependent on the proton motive force (pmf) mainly produced directly by respiratory complex I and indirectly by the respiratory complex II, respectively encoded by the *nuo* and *sdh* operon [12,15,29]. Thus, based on our previous study [7], one hypothesis was that RyhB induced resistance was due to an inhibitory effect on the activity of these two complexes that would block entry of gentamicin in the cell.

**Fig 2:**
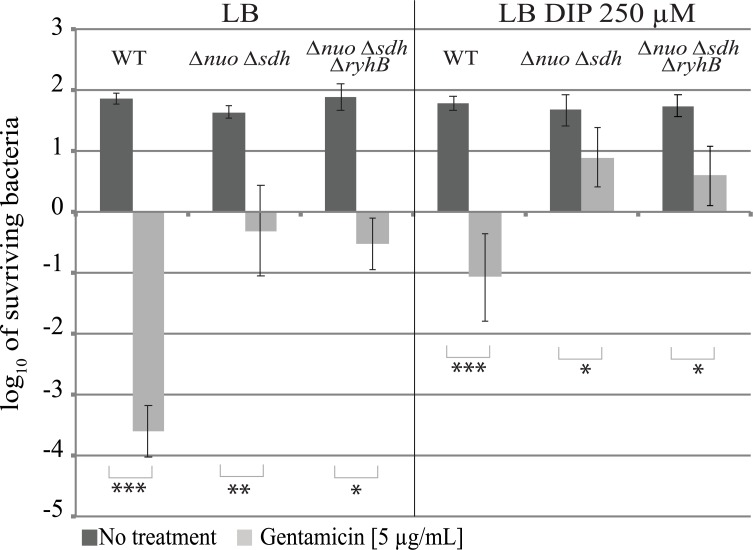
RyhB induced gentamicin resistance is dependent on Nuo and Sdh. The *Δnuo Δsdh* (BEFB20) and *Δnuo Δsdh ΔryhB* (SC024) strains were grown for 3 h with or without gentamicin (5 μg / mL) in LB (left panels) or in LB with DIP 250 μM (right panels). Colony forming units were counted to determine the number of surviving bacteria. Points were normalized relatively to t0 and plotted as log_10_ of surviving bacteria. The absolute c.f.u. at time-point zero was ≈ 5.10^7^ c.f.u. / mL for each sample. Error bars represent the standard deviations of three independent experiments. Statistical analyses were performed with Student’s T-test: *p < 0,05; **p < 0,01.

To test this hypothesis killing assays were run with a strain deleted for both respiratory complexes (*Δnuo Δsdh*). As expected, this mutant was resistant to gentamicin (Fig 2, left panel) [7]. Adding DIP to the medium somewhat increased by 1 log the survival of the *nuo sdh* mutant, suggesting that pmf might be even more decreased in these conditions. Nevertheless, deleting *ryhB* from this strain did not increase its sensitivity to gentamicin during iron starvation (Fig 2, right panel) indicating that the observed DIP enhancing sensitivity of the *ryhB* mutant was dependent on *nuo* and *sdh*.

We further assessed the implication of each of the respiratory complexes by testing the sensitivity of the *Δnuo* and *Δsdh* single mutants, deleted or not for *ryhB* (S3 Fig). The *nuo* simple mutant was almost completely resistant to gentamicin in presence of DIP, whether *ryhB* was present or not. In contrast, the *sdh* simple mutant became somewhat more sensitive (1 log) when *ryhB* was deleted from the chromosome. We conclude from these results that while both complexes are needed for full sensitivity of *ryhB* mutants to gentamicin, Nuo seems to be slightly more important than Sdh.

### RyhB represses the activity of the respiratory complexes

**Fig 3:**
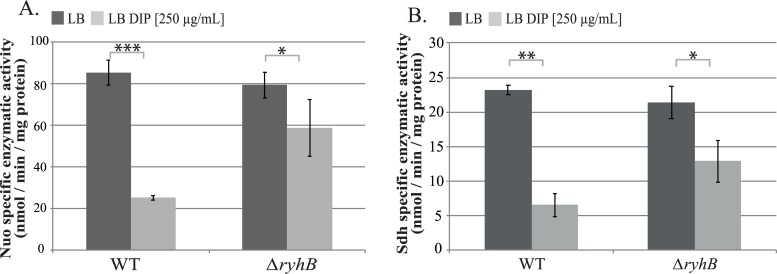
RyhB decreases Nuo and Sdh enzymatic activities. A: NADH specific enzymatic activity of Nuo in WT or *ΔryhB* strain grown in LB (dark grey bars) or in LB containing DIP (light grey bars) were determined by following the disappearance of the D-NADH substrate by spectrophotometry (nmol / min / mg protein). B: Succinate dehydrogenase activities in WT or *ΔryhB* strains grown in LB (dark grey bars) or in LB containing DIP (light grey bars) were determined by following the absorbance of DCPIP (nmol / min / mg protein). Bars represent the mean of at least three experiments and error bars represent the standard deviations. Statistical analyses were performed with Student’s T-test: *p < 0,05; **p < 0,01; ***p < 0,001.

Results above suggested that RyhB inhibits the activity of both respiratory complexes during iron starvation. To test this, we measured Nuo and Sdh specific enzymatic activities in WT and *ryhB* mutant strains grown in the presence or absence of the iron chelator DIP. In the WT strain grown in the presence of DIP, Nuo activity was reduced down to 25% as compared with the WT grown in the absence of DIP (Fig 3A). In contrast, Nuo activity was only modestly reduced in the *ryhB* mutant grown in presence of DIP. The same pattern was also observed for Sdh activity (Fig 3B). Altogether, these results confirm that RyhB represses the activities of both Nuo and Sdh complexes in medium deprived for iron.

### RyhB represses *nuo* and *sdh* expression

RyhB inhibition of Sdh and Nuo activities may be due to the repression of the synthesis and / or of the maturation of the complexes. Expression of *sdh* has already been shown to be repressed by RyhB [20,30]. In contrast, although pointed out in global approaches, RyhB regulation of *nuo* genes expression still awaited investigation [17,31–33].

**Fig 4:**
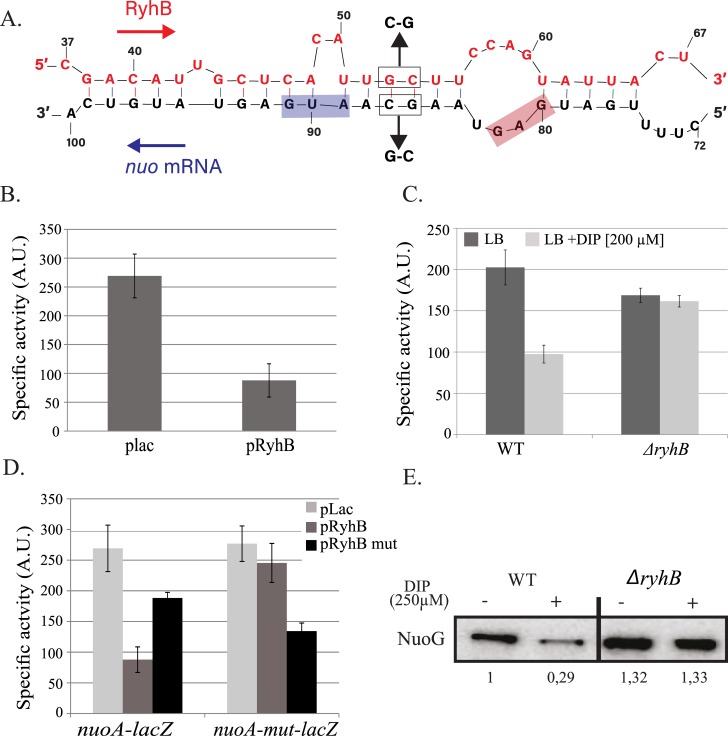
RyhB represses *nuo* expression. A: base-pairing predicted between RyhB and *nuo* mRNA. Nucleotides belonging to *ryhB* are represented on top, those corresponding to *nuo* on the bottom. Relative position to the transcriptional start site of *ryhB* and *nuo* are indicated above and below the sequences, respectively. B: the SC005 strain containing a P_BAD_-*nuoA-lacZ* fusion was transformed with the empty plac vector or with the pRyhB plasmid containing *ryhB* under the control of an IPTG inducible promoter. Cells were grown in LB containing ampicillin (25 μg/mL), IPTG (100 μM) and arabinose (0,02%) during 6 h after which ß-galactosidase activity was determined. Specific activities are represented by arbitrary units that were empirically determined to be approximately equivalent to Miller units. Error bars represent the standard deviations of six independent experiments. C: strains containing the P_BAD_-*nuoA-lacZ* fusion, WT (SC005) or deleted for *ryhB* (SC006) were grown in LB with or without DIP (200 μM) during 6h before ß-galactosidase activities were measured. Each bar represents the mean from six independent experiments; error bars represent the standard deviations. D: Strains containing either the P_BAD_-*nuoA-lacZ* or the P_BAD_-*nuoA*_*mut*_*-lacZ* fusions were transformed with the plac, pRyhB or pRyhBmut plasmids and ß-galactosidase activity were determined. Each point represents the mean from six or more experiments. E: WT and *ryhB* mutant cell extracts from cultures grown in LB or in LB with DIP (250 μM) were subjected to immunoblot analyses using antibodies raised against NuoG. Quantification represents the mean of three different experiments.

Using the RNA-fold software (http://unafold.rna.albany.edu), we could predict a base-pairing in between RyhB and the 5’ un-translated region of the first gene of operon, *nuoA* [34]. This base-pairing involves 21 nucleotides (nt) of RyhB and includes the ribosome-binding site (RBS) and the start codon of *nuoA* (Fig 4A). Overexpression of *ryhB* on a plasmid decreased the activity of a P_BAD_-*nuoA-lacZ* fusion of about 4-fold, as compared to cells transformed with an empty vector (Fig 4B). In addition, the P_BAD_-*nuoA-lacZ* activity was decreased by 2-fold when WT cells were treated with DIP. This was in sharp contrast with the isogenic *ryhB* mutant strain for which activity remained the same in presence or absence of DIP (Fig 4C).

We then tested the biological relevance of the predicted base-pairing by introducing point mutations in the P_BAD_-*nuoA-lacZ* chromosomal fusion, giving rise to the *nuoA*_*mut*_*-lacZ* fusion (G86C and C87G; Fig 4A). In contrast to the WT *nuoA-lacZ* fusion, RyhB overexpression was no longer able to repress activity of the *nuoA*_*mut*_ fusion (Fig 4D). We then introduced compensatory mutations in the pRyhB plasmid that should restore base-pairing to the mutated, but not to the WT, *nuo-lacZ* fusion, giving rise to pRyhB_mut_. As seen in Fig 4D, overexpression of RyhB_mut_ failed to fully repress the WT *nuo-lacZ* fusion but was able to repress *nuoA*_*mut*_*-lacZ* fusion. Altogether these results strongly suggest that RyhB represses *nuo* expression by base-pairing on the mRNA upstream *nuoA*.

We then evaluated the effect of this repression on protein levels by performing Western blot analyses against NuoG, a protein of the complex. Strikingly, NuoG protein levels decreased steeply, about 3-fold, when the WT strain was grown in presence of DIP (Fig 4E). This phenotype was suppressed in the *ryhB* mutant, confirming the *in vivo* inhibition of Nuo synthesis by RyhB.

As a control and to compare *sdh* regulation to *nuo*, we performed a series of similar tests on a *sdhC-lacZ* fusion. We saw that RyhB overexpression repressed the expression of the fusion by more than 10-fold (S4A Fig). In addition, the WT fusion was also strongly inhibited when cells were grown in presence of DIP but not when *ryhB* was deleted (S4B Fig). Identical conclusions were reached from analyzing SdhB protein levels by performing Western blots (S4C Fig). These experiments thus confirm the regulation of *sdh* by RyhB.

### RyhB may impact Nuo and Sdh maturation through *iscSUA* repression

Biogenesis of Fe-S clusters by the Isc machinery has been shown to be key for full Nuo and Sdh activity and their associated pmf production. The *iscSUA* mRNA is a known RyhB target [16]. Therefore, we asked if RyhB-mediated repression of the *iscSUA* genes bears any consequence on maturation of Nuo and Sdh.

We first checked that RyhB repressed *isc* expression in our conditions by following levels of the IscS protein after treatment with DIP in a WT and in a *ryhB* mutant strain (S5 Fig). Interestingly, DIP treatment did not seem to affect levels of IscS in the WT strain. However, in agreement with the study of Desnoyer et al. [16], levels of IscS rose by a two-fold factor in the *ryhB* mutant after 90 minutes of DIP treatment. These results thus confirm that RyhB limits the expression of Isc during iron starvation, albeit modestly, counteracting IscR alleviation of repression at the level of the P*isc* promoter.

**Fig 5:**
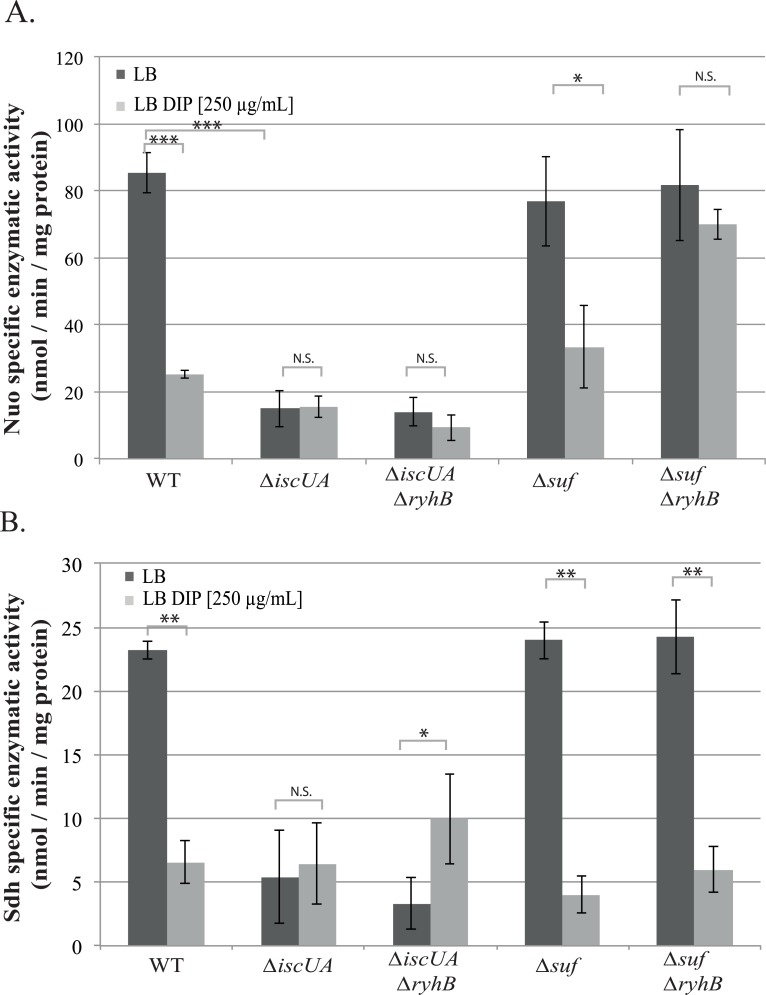
RyhB inhibits Nuo enzymatic activity by repressing *isc*. Nuo (A) and Sdh (B) specific enzymatic activities of *Δisc* and *Δsuf* mutants containing or not *ryhB* grown in LB (dark grey bars) or in LB containing DIP (light grey bars) were determined. Bars represent the mean of 3 independent experiments and error bars represent the standard deviations. Statistical analysis were performed with Student’s T-test: *p < 0,05; **p < 0,01; ***p < 0,001; N.S.: Not significant.

We then measured Nuo and Sdh specific activities in strains deleted for *suf* (deletion of the whole operon) or for *isc* (*iscUA* deletion mutant), with or without *ryhB*. We first checked that deleting *ryhB* from the *isc* and *suf* mutants did not perturb growth in presence of DIP (S6 Fig). Growth of the *iscUA* mutant in LB was slightly slower than the WT strain, as expected from the literature, and was not affected by adding DIP to the medium (S6A Fig) [35]. Introducing a secondary *ryhB* mutation did not change growth of the *iscUA* mutant. In sharp contrast, growth of the *suf* mutant was severely affected in presence of DIP (S6B Fig). This was expected since the *suf* mutant was shown to be essential for growth in defined media containing higher doses of DIP (≥300 μM) [36]. Interestingly, introducing a *ryhB* mutation in the *Δsuf* background slightly improved growth, perhaps suggesting that the alleviation of *isc* repression in the *suf* mutant may partially restore Fe-S cluster homeostasis. We then measured Nuo activities of the different mutants grown until mid-exponential phase (OD_600_ = 0,6). In agreement with the literature [7], Nuo activity was decreased more than 5 fold in an *isc* mutant wherein the Suf machinery alone is responsible for Fe-S biogenesis (Fig 5A). Nuo activities of the *isc ryhB* mutant remained low in iron-deprived conditions.

Nuo activity of the *Δsuf* strain was comparable to that of the WT and DIP treatment inflicted the same drop inactivity in both strains. Strikingly however, deleting *ryhB* in the *Δsuf* mutant almost completely restored Nuo activity of cells grown in low iron condition. These data strongly suggest that the contribution of Isc to maturation of Nuo complex is of paramount importance, even in growth conditions limited in iron availability.

The situation was slightly different for Sdh. Deleting *isc* severely affected activity of Sdh in presence or absence of iron. Further deleting *ryhB* from this strain marginally restored Sdh activity, indicating that a significant level of Sdh maturation can be controlled by Suf. In sharp contrast to Nuo however, activity of Sdh was not restored when *ryhB* was deleted in the *suf* mutant (Fig 5B). These results thus suggest that Isc cannot ensure maturation of Sdh in low iron conditions.

### There is de novo synthesis and maturation of the Nuo respiratory complex in the *ryhB* mutant during iron starvation

The fact that we could see Nuo activity during iron starvation in the *ryhB* mutant was surprising as it suggested that there is Isc dependent de novo biogenesis of Fe-S clusters, at least for this complex, in iron limiting conditions. To further test this hypothesis, we measured Nuo activity in cells treated with DIP (250 μM) for one hour.

**Fig 6:**
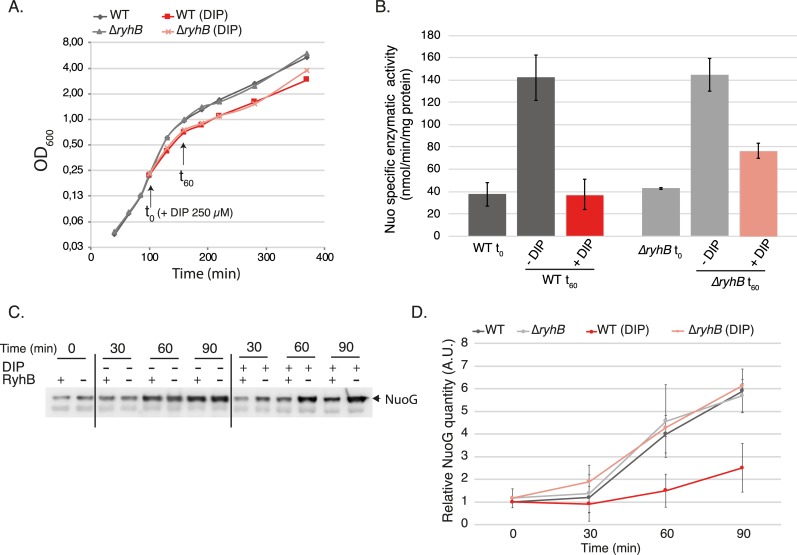
De novo synthesis and maturation of Nuo complex during iron starvation. A: WT (dark lines) or Δ*ryhB* strain (light lines) were grown in LB until they reach OD_600_ = 0,2 (t_0_) 250 μM of DIP was then added to the cultures (red lines) or not (grey lines). Growth curves were determined by following the absorbance at 600 nm over time. Growth curves represented here are representative of three experiments. B: NADH specific enzymatic activies of total extracts of the WT or Δ*ryhB* strain over time. Strains were grown as in (A) and extracts were taken at t_0_ (OD_600_ = 0,2) and t_60_. Activities of cells cultivated with (red bars) or without (grey bars) DIP were determined by following the disappearance of the D-NADH substrate by spectrophotometry (nmol / min / mg of total proteins in the extract). Bars represent the mean of three independent experiments and error bars represent the standard deviations. C: WT and ryhB mutant cell extracts from cultures grown as in (A) in LB until 0,2 (t_0_) then incubated in LB containing 250 μM DIP or not during 90 min were subjected to immunoblot analyses using antibodies raised against NuoG. D: quantification of immunoblots as realized in C. Quantifications were made using the Image J program, using an unspecific band as a loading control, and were relativized to the WT strain at t_0_ (set to 1). Points represent the mean of three independent experiments and error bars represent the standard deviation.

Treatment with DIP induced a slight delay in growth that was identical for both WT and *ryhB* mutant cells (Fig 6A). In untreated cells, Nuo activity increased with growth as already reported (Fig 6B) [37]. In sharp contrast, WT cells treated with DIP for one hour did not show any increase in Nuo activity, while in the *ryhB* mutant Nuo activity showed a two-fold increase (40 to 80 nmol /min /mg of protein). Likewise, levels of NuoG protein increased in *ryhB* mutant cells treated with DIP, but not in the WT strain (Fig 6C and 6D). These experiments thus support the idea that both de novo synthesis and maturation of Nuo take place in *ryhB* cells treated with DIP.

Incidentally, we note that while there is a four-fold increase in the quantity of Nuo proteins in *ryhB* mutant cells after one-hour treatment, there is only a two-fold increase in Nuo activity. This thus strongly suggests that while Isc is able to ensure maturation of Nuo in iron depleted conditions, it is not as efficient as in iron replete conditions.

### RyhB induces gentamicin phenotypic resistance by repressing *isc*, *nuo* and *sdh* expression

In order to better appraise the role of Fe-S clusters maturation inhibition by RyhB in the resistance to gentamicin, we performed sensitivity assays in strains containing only one of the two Isc or Suf Fe-S biogenesis machineries.

**Fig 7:**
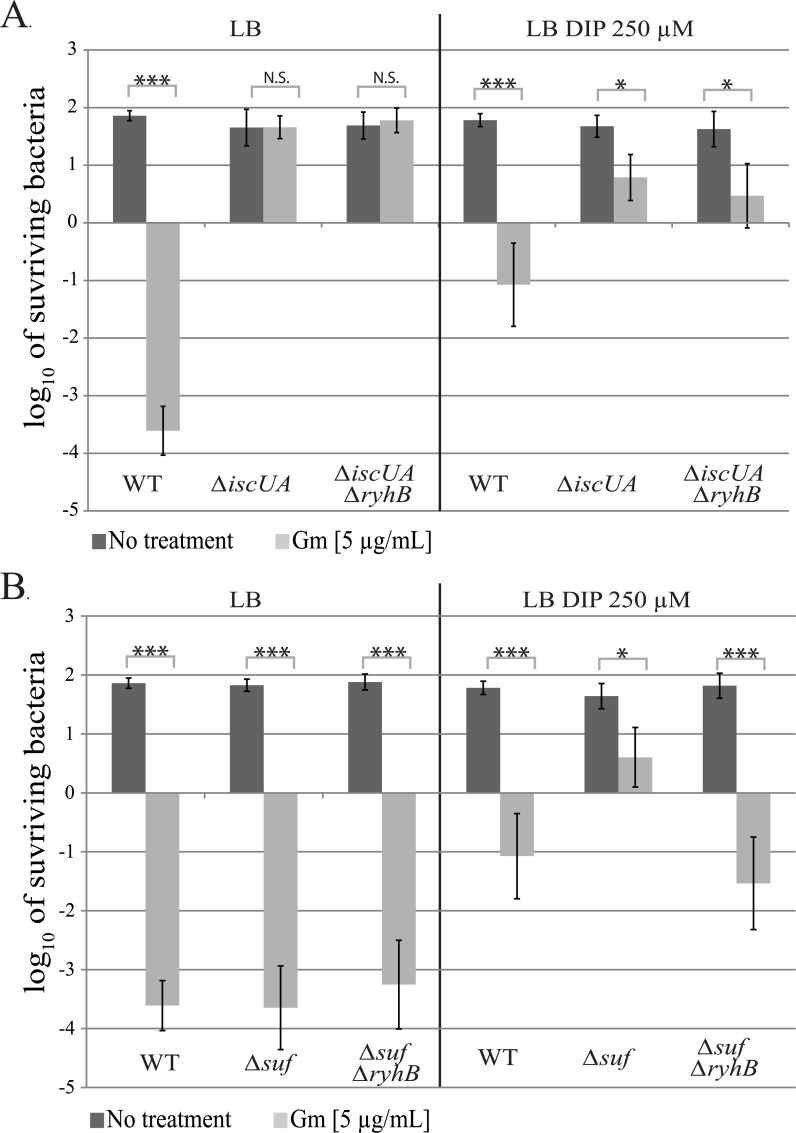
RyhB induces gentamicin resistance by inhibiting Fe-S clusters maturation. The *Δisc* (A) and the *Δsuf* (B) strains containing or not *ryhB* were grown with (light grey bar) or without (dark grey bars) gentamicin (5 μg/mL) for 3 h in LB (left panels) or in LB with DIP (250 μM) (right panels). After that, cells were diluted in PBS and spotted on LB agar plates. c.f.u. and Log_10_ of surviving bacteria numbers were determined. Error bars represent the standard deviation of three independent experiments. Statistical analyses were performed with Student’s T-test: *p < 0,05; ***p < 0,001; N.S.: Not significant.

As previously shown, the *isc* mutant was fully resistant to gentamicin in LB (Fig 7A) [7]. This phenotype remained unchanged when DIP was added to the medium, whether RyhB was present or not (Fig 7A), thus showing that the slight Sdh activity observed in these conditions (Fig 5) is not sufficient to render the cells sensitive to gentamicin. In sharp contrast, introducing a *ryhB* mutation restored sensitivity of a *suf* mutant strain when grown in presence of DIP (Fig 7B), which is in agreement with the restoration of Nuo activity in this strain under these conditions.

As Nuo and Sdh activities are crucial for gentamicin sensitivity, we investigated if we could correlate both the levels of complexes enzymatic activity with that of resistance to gentamicin. Strikingly, there was an almost linear correlation between Nuo or Sdh activities of each strain and its sensitivity to gentamicin (S7A and S7B Fig). For instance, strains displaying the lowest Nuo activities were the most resistant to gentamicin, and vice versa.

## Discussion

**Fig 8:**
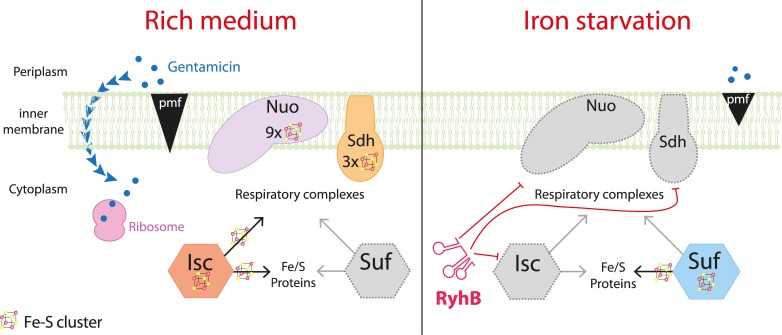
Model for the RyhB induced resistance to gentamicin during iron starvation. When iron is not limiting (left panel), the Isc Fe-S biogenesis machinery ensures the maturation of Nuo and Sdh, which generate a pmf that allows gentamicin uptake. Gentamicin reaches the ribosome and induces mistranslation, which renders cells sensitive to the antibiotics. When iron is scarce (right panel), RyhB is expressed and represses the expression of *nuo*, *sdh* and *isc*. The pmf is lowered and gentamicin cannot enter the cytoplasm thus making cells resistant to the antibiotic.

Phenotypic resistance can take place when environmental conditions change as adaptive molecular responses modify cellular physiology, giving rise to a transient resistance state. Here, we show that the sRNA RyhB is a major contributor of *E*. *coli* phenotypic resistance to gentamicin in iron limiting conditions. Aminoglycosides uptake depends upon pmf, which is produced by the activity of respiratory complexes I (Nuo), and, indirectly, by complex II (Sdh). RyhB negatively regulates synthesis of both respiratory complexes. RyhB may also impact activity of Nuo indirectly by limiting the levels of Isc, which we show to be essential for its maturation (i.e. acquisition of Fe-S clusters) (Fig 8). Our model strengthens the role of the pmf-producing respiratory complexes in entry of aminoglycosides. Fe-S biogenesis maturation of the complexes was earlier pointed out as the main factor for resistance [7]. By identifying here that the *nuo* mRNA is targeted by RyhB in addition to *sdh*, we show that synthesis of the respiratory complexes is also key in this process.

As early as 2005, the *nuo* mRNA was suspected to be a target of RyhB as the operon was down-regulated when the sRNA was over-expressed, [17]. The *nuo* mRNA was also more recently found associated with Hfq and RyhB in a global study of sRNA-mRNA interactions [33]. We here could predict and confirm a direct base-pairing of RyhB to the *nuo* mRNA at the level of the UTR of *nuoA*, the first gene of the operon. This base-pairing occurs close to the ribosome binding site of *nuoA*, which strongly suggests that RyhB represses expression of *nuo* by occluding binding of the ribosome, and subsequently degradation of the mRNA [38]. The *nuo* mRNA is very long (about 15 kb) and comprises 14 genes, which makes it one of the longest mRNAs regulated by a sRNA to our knowledge. Importantly, in addition to the effects seen on *nuoA* expression by using beta-galactosidase assays as a read-out (Fig 4), we could also observe repression at the level of the NuoG protein by using Western blots analysis (Fig 4E). The structural *nuoG* gene lies more than 5 kb away from the RyhB/Nuo base-pairing site. It will thus be interesting to investigate how far downstream the *nuo* operon RyhB repression propagates.

Respiratory complexes are high iron consumers, with a total of 12 Fe-S clusters for Nuo and Sdh in *E*. *coli*. Thus, their repression by RyhB is in line with its role in installing an iron sparing response when iron becomes scarce [17,19]. Before our results, one could have imagined that RyhB represses Nuo and Sdh expression in order to limit accumulation of inactive apo-complexes in iron scarce conditions. However, both protein levels and activity of Nuo are restored in a *ryhB* mutant in iron-deprived medium indicating that maturation of respiratory complex I is possible under these conditions. These results strongly suggest that RyhB inhibits synthesis of Nuo Sdh to preclude respiratory complexes to divert iron from other essential processes.

We here show that Isc is essential for Nuo maturation when iron is depleted in the *ryhB* mutant (Fig 3A and Fig 5A). In contrast, maturation of Sdh was only partially restored in the *ryhB* mutant in presence of DIP (Fig 3B) and, perhaps more surprisingly, this activity did not seem to be dependent on Isc but rather on Suf (Fig 5B). More investigation is needed to understand the molecular basis for the difference in between Isc and Suf substrates preference. In any case, our results also clearly show that Nuo activity is more important than that of Sdh in installing a phenotypic resistance to gentamicin (S2 Fig). This may relate to pmf production by Nuo and Sdh. Indeed, Nuo, but not Sdh, directly translocates 4 protons across the membrane while both indirectly contribute to pmf production by passing electrons to cytochrome oxidase [12,29].

Our experiments clearly show that there is de novo synthesis and biogenesis, at least of Nuo complexes, during iron starvation in the *ryhB* mutant (Fig 6). We also confirm that the maturation of this respiratory complex depends on the Isc machinery. Taken together, these results indicate that the Isc machinery can be functional during iron starvation and mature at least the Nuo complex. In agreement with previous results from the Massé laboratory [16], we also show that RyhB limits levels of the Isc machinery during iron starvation. Thus, a tempting hypothesis is that RyhB inhibits indirectly Nuo activity by limiting its maturation by Isc. However, given that RyhB effect on Isc is relatively modest, we cannot exclude that producing Nuo complexes alone, even while keeping Isc synthesis repressed by RyhB, may be sufficient to restore Nuo activity. Furthermore, the situation might be even more complex as iron depletion is likely to modify to different extent both the levels and activity of all of the proteins intervening in this process, namely Isc, Suf, Nuo and Sdh. Thus, fully testing the hypothesis that RyhB-mediated reduction of Isc synthesis will bear an effect on Nuo/Sdh activity will require a thorough assessment of both the concentration and the activity of all components cited above. For the time being, we consider the hypothesis of an indirect control of Nuo maturation as a likely contribution to the RyhB dependent phenotypic resistance we observed.

Nevertheless, the fact that Isc is able to maturate Nuo in iron deprived conditions may seem contradictory with previous studies that have shown the Suf system to be essential during iron starvation [36]. In agreement with that idea, we have seen that deleting *ryhB* partially suppressed the growth defect phenotype of a *suf* mutant grown in the presence of DIP (S6 Fig). However, growth of this mutant is not restored to wild type-like levels, indicating that while when overexpressed Isc may promote Fe-S cluster biogenesis, during iron starvation, it is not as efficient as the Suf machinery and thus explaining the need for a second iron limitation stress responsive system.

Our study puts RyhB on the focus among a growing number of sRNAs that have been directly or indirectly linked to antibiotic resistance [39–41]. However, in most of these cases phenotypes were derived from overexpression of the sRNAs and such situations might not be relevant to physiological conditions. For instance, 17 out of 26 *E*. *coli* sRNAs that were assessed in a systematic manner against a variety of antibacterial effectors were shown to affect sensitivity to antibiotics when overexpressed, but few showed any phenotype when mutated [42].

A most spectacular case is represented by the role RyhB could play in the bacterial persistence of uropathogenic *E*. *coli* to different classes of antibiotics, among which gentamicin [43]. Persistence is a phenomenon in which a fraction of the bacterial population enters a metabolically inactive state that enables it to survive exposure to bactericidal antibiotics [44]. It was proposed that *ryhB* mutants would induce less persister cells because they display increased ATP levels and altered NAD^+^ / NADH ratios. In the light of our results, we believe these effects could also be explained by the fact that *ryhB* mutants display higher levels of Nuo, Sdh and Isc and therefore are more metabolically active, but also more prone to uptake the antibiotic. It is noteworthy that these experiments were conducted in rich medium not devoid for iron, and after long treatment with antibiotics (four days), which may explain low induction of RyhB in only a small percentage of bacterial cells that would then be able to resist antibiotics treatment in a persister-like manner.

RyhB homologs and paralogs are found in multiple other bacterial species, which suggests that many bacteria outside of *E*. *coli* may share the resistance mechanism that we describe here [45]. In particular, other pathogenic bacteria such as *Yersinia*, *Shigella* or *Salmonella* possess not only RyhB homologs, but also the Isc and Suf system and rely on Nuo and Sdh for respiration on oxygen [46,47]. RyhB has also been implicated in promoting sensitivity to colicin IA, which is not an antibiotic in a narrow sense, but a bacteriocin secreted by other species to outcompete bacteria sharing the same niches [48]. In addition, RyhB has been shown to be involved in the virulence of *Shigella dysenteriae* by repressing the major virulence regulator *virB*, and the sRNA may be associated with the virulence of *Yersinia pestis*, as the expression of its two RyhB homologs (RyhB1 and RyhB2) increases in the lung of infected mice [49,50]. Altogether, these data point out for a major role for RyhB in escaping antibacterial action.

## Materials and methods

### Strains and culture

All strains used in this study are derivatives of *E*. *coli* MG1655 and are listed in S1 Table. Strains were grown in LB broth (Difco), containing various concentrations of 2,2’-dipyridyl (DIP) (Sigma) when stated. Transductions with P1 phage were used for moving marked mutation as described previously in [51]. The plac and pRyhB plasmids used in this study are described and have been transformed as previously described in [52]. All oligonucleotides used are listed in S2 Table.

### Antibiotic sensitivity experiments

Starting from overnight cultures in LB, strains were diluted 1/100 time in fresh medium containing or not DIP and grown aerobically at 37°C with shaking until OD_600_ ≈ 0.2. At this point, antibiotics were added to the cells (gentamicin: 5 μg / mL; ampicillin: 5 μg / mL; tetracycline: 5 μg / mL and norfloxacin: 25 ng / mL). After 3 h cells were taken, diluted in PBS buffer and spotted on LB agar plates and incubated at 37°C for 16 h. Cell survival was determined by counting the number of colony-forming units per mL (c.f.u. / mL). The absolute c.f.u at time-point 0 was of ≈ 5 x 10^7^ cells / mL in all experiments.

### Minimum inhibitory concentration (MIC) determination

The MIC were determined as previously described [53]. Briefly, each antibiotic containing-well (with 0; 2,5; 3,75; 5; 6,25; 7,5; 8,75; 10; 11,25; 12,5; 13,75; 15 μg / mL of gentamicin respectively) of a 96-well micro-titer plate was inoculated with 100 μL of a fresh LB bacterial inoculum of 2 × 10^5^ c.f.u / mL. The plate was incubated at 37°C for 18 h under aerobic conditions. OD_600_ for each well was then determined by measuring the absorbance on a Tecan infinite 200. MIC was defined as the lowest drug concentration that exhibited complete inhibition of microbial growth.

### Fusions construction

The P_BAD_*-nuoA-lacZ* and P_BAD_-*sdhC-lacZ* fusions were constructed and recombined in PM1205 strain, as previously described [25]. Briefly, sequences corresponding to *nuo or sdh* genes starting from its +1 transcriptional start up to 30 nucleotides downstream of the ATG codon were amplified using oligonucleotides P_BAD_-nuoA-F or P_BAD_-sdhC-F, and lacZ-nuoA-R or lacZ-sdhC-R, respectively. PCR amplifications were carried out using the EconoTaq DNA polymerase from Lucigen. The purified PCR products were then electroporated into strain PM1205 for recombination at the *lacZ* site. Recombinants carrying the desired fusions (SC005 and SC009) were selected on LB plates devoid of NaCl and containing 5% sucrose, 0,2% arabinose and 40 μg / mL X-Gal (5-bromo-4-chloro-3-indolyl-D-galactopyranoside). Blue colonies were chosen, and the resulting fusions were sequenced using oligonucleotides lacI-F and Deep-lac.

Overlap PCR was used to introduce point mutation in the fusion. The two PCR products corresponding to the sequence upstream and downstream of the desired mutation were amplified by PCR with oligonucleotides nuoAmut-F and Deep-lac, and LacI-F and nuoAmut-R containing the desired mutation and using genomic DNA from the SC005 strain as a template. The two PCR products were then joined by an overlap PCR using oligonucleotides lacI-F and Deep-lac. The resulting PCR products were purified, electroporated in strain PM1205 and sequenced as described above.

For point mutations in the pRyhB plasmid, the pRyhB plasmid was first purified from a WT (dam^+^) *E*. *coli* strain, and then amplified by PCR with oligonucleotides RyhBmut-F and RyhBmut-R, containing the desired mutation. The native plasmid was eliminated from the resulting PCR product by Dpn1 enzyme digestion for 1 h at 37°C. Plasmids containing the desired mutation were then purified and transformed in SC005 and SC0026 strains.

### β-galactosidase experiments

Overnight cultures of different strains were diluted 1/100 times in fresh medium in culture flasks containing ampicillin and IPTG (isopropyl ß-D-1thiogalactopyranoside) or DIP when indicated. After ≈ 7 hours of growth 100 μL of cultures were dispatched in 96 wells microtiter plates (triplicates for each conditions). Absorbance at 600 nm was measured in a microtiter plate reader (Tecan infinite 200). Then, 50 μL of permeabilization buffer were added in each well (100 mM Tris HCl pH 7,8; 32 mM Na_2_HPO_4_; 8 mM EDTA; 40 mM Triton) and the microtiter plate was incubated for 10 minutes at room temperature. O-Nitrophenyl-β-D-galactopyranoside (ONPG) was added to the solution and appearance of its degradation product was immediately determined by measuring the absorbance at 420 nm on a Tecan infinite 200 during 30 minutes. The specific activities were calculated by measuring the Vmax of the OD_420_ appearance divided by the OD_600_. Values were then multiplied by 100000, a coefficient that was chosen empirically to approximate Miller units.

### Nuo and Sdh enzymatic activities

The Nuo and Sdh enzymatic activities were determined as previously described [54,55]. Briefly, overnight cultures of the strains of interest were diluted 1/100 times in fresh LB medium containing or not 250 μM of DIP and grown at 37°C with shaking until they reached OD_600_ ≈ 0.6. Cultures were pelleted by centrifugation (11 000 G, 10 min at 4°C) and washed in phosphate buffer (50 mM pH 7,5). Cells were then lysed at the French press and 100 μL were immediately frozen in liquid nitrogen before determining Nuo activity. Nuo enzymatic activity was determined at 30°C by monitoring the disappearance of the specific Deamino-NADH (DNADH) substrate at 340 nm every 5 s during 10 min at 30°C in a spectrophotometer.

For Sdh activity determination, lysate samples from French press were pellet by centrifugation (11 000 G, 10 min at 4°C) and the supernatant was used for membrane fraction preparation by ultracentrifugation at 45 000 G at 4°C during two hours. Pellets were then resuspended in phosphate buffer and kept in liquid nitrogen for later Sdh activity measurements. The enzyme was first activated by incubation in 50 mM Tris-HCl (pH 7.5), 4 mM succinate, 1 mM KCN for 30 min at 30°C. The enzymatic activity was measured in the membrane fraction by monitoring Phenazine EthoSulfate (PES)-coupled reduction of dichlorophenol indophenol (DCPIP) at 600 nm, in a reaction containing 50 mM Tris-HCl (pH 7.5), 4 mM succinate, 1 mM KCN, 400 μM PES and 50 μM DCPIP.

The specific activities were calculated by measuring the Vmax divided by the protein concentration in total extracts evaluated by absorbance at 280 nm.

### Quantification of Nuo, Sdh and Isc protein levels by Western blot analyses

Total extracts and membranes preparation prepared for Nuo and Sdh activities were used for quantification of Nuo and Sdh protein levels, respectively. Total protein levels were determined by measuring absorbance at 280 nm on a spectrophotometer. Same amount of total protein level were migrated on poly-acrylamide gels Tris-gly Sodium Dodecyl Sulfate (Novex 4–20% Tris-Glycine Mini Gels) then, transferred on nitrocellulose membrane using Pierce G2 Fast Blotter (25 V, 1,3 mA, 7 min). Protein level were detected by incubating the membrane with α-NuoG, α-SdhB, or α-IscS (1/1000) antibodies from rabbit and then by an α-rabbit antibody (1/1000) coupled with Hrp peroxidase. Signals were detected by chemiluminescence with Pierce ECL Western blotting system on an ImageQuant LAS 4000 camera. Quantification of protein levels was determined by measuring the specific signal intensity of the bands corresponding to Nuo, Sdh or IscS proteins with the ImageJ software. Intensities were normalized using an unspecific band detected by the same antibody.

## Supporting information

S1 Fig Growth of the WT and *ryhB* mutant in presence of dipyridyl. A: Growth curves of WT (dark lines) or ΔryhB strain (light lines) in LB (grey lines) or in LB containing 250 μM DIP (red lines) were determined by following the absorbance at 600 nm. Error bars represent the standard deviations of three independent experiments. B: Doubling time of WT and Δ*ryhB* strains calculated from the growth curves measurements in (A).
(EPS)

S2 Fig RyhB increases the resistance to gentamicin during iron starvation. The WT and the *ΔryhB* mutant MIC were determined by growing cells in medium containing various concentrations of gentamicin and the iron chelator DIP (250 μM). The MIC was defined as the lowest drug concentration that exhibited complete inhibition of microbial growth. Statistical analysis were performed with Student’s T-test: **p < 0,01; N.S.: Not significant.
(EPS)

S3 Fig Sensitivity of *nuo* and *sdh* single mutants to gentamicin. *Δnuo* (BEFB05), *Δnuo ΔryhB* (SC085), *Δsdh* (BEFB06) and *Δsdh ΔryhB* (SC086) strains were grown with or without gentamicin (5 μg / mL) for 3 h in LB with DIP 200 μM. Colony forming units were counted to determine the number of surviving bacteria. Points were normalized relatively to t0 and plotted as log_10_ of surviving bacteria. The absolute c.f.u. at time-point zero was ≈ 5.10^7^ c.f.u. / mL for each sample. Error bars represent the standard deviation of three independent experiments. Statistical analysis were performed with Student’s T-test: *p < 0,05; **p < 0,01; N.S.: Not significant.
(EPS)

S4 Fig RyhB represses *sdh* expression. A: strain containing a P_BAD_-*sdhC-lacZ* fusion (SC009) was transformed with the empty plac vector or with pRyhB plasmid containing *ryhB* under the control of an IPTG inducible promoter. Cells were grown in LB containing ampicillin (25 μg/mL), IPTG (100 μM) and arabinose (0,02%) during 6 h after which ß-galactosidase activity was determined. Specific activities are represented by arbitrary units that were empirically determined to approximate Miller units. Error bars represent the standard deviations of six independent experiments. B: strains containing P_BAD_-*sdhC-lacZ* WT (SC009) or deleted for *ryhB* (SC010) were grown in LB with or without DIP (200 μM) during 6h before ß-galactosidase activities were measured. Each bar represents the mean from six independent experiments. C: WT and *ryhB* mutant cell extracts from cultures grown in LB or in LB with DIP (250 μM) were subjected to Western blot analyses using antibodies raised against SdhB. Quantification represents the mean of three different experiments.
(EPS)

S5 Fig IscS protein levels increase in the *ryhB* mutant in presence of dipyridyl. WT and *ryhB* mutant cell extracts from cultures grown in LB until 0,2 (t0) then incubated in LB containing 250 μM DIP, or not, during 90 min were subjected to immunoblot analyses using antibodies raised against IscS. Values behind each bar represent the relative quantification of IscS protein over three experiments. Quantifications were made using the Image J program, using an unspecific band as a loading control, and were relativized to the WT strain at t0 (set to 100). Values in between parenthesis represent the standard deviation.
(EPS)

S6 Fig Growth of the *isc* and *suf* mutants derivatives in presence of dipyridyl. A: Growth curves of Δ*isc* (dark lines) or Δ*isc* Δ*ryhB* strain (light lines) grown in LB (grey lines) or in LB containing 250 μM DIP (red lines) were determined by following the absorbance at 600 nm over time. Error bars represent the standard deviations of three independent experiments. B: Growth curves of Δs*uf* (dark lines) or Δ*suf* Δ*ryhB* strain (light lines) grown in LB (grey lines) or in LB containing 250 μM DIP (red lines) were determined during by following the absorbance at 600 nm over time. Error bars represent the standard deviations of three independent experiments. C: Doubling time of Δ*isc*, Δ*isc* Δ*ryhB*, Δ*suf* or Δ*suf* Δ*ryhB* strains calculated from the growth curves in A and B.
(EPS)

S7 Fig Gentamicin sensitivity can be directly correlated with Nuo and Sdh specific activities. Sensitivity to gentamicin of WT, Δ*ryhB*, Δ*isc*, Δ*isc* Δ*ryhB*, Δ*suf* and Δ*suf* Δ*ryhB* strains grown in LB (black points) or in LB containing DIP (red points) were plotted relatively to their Nuo (A) or Sdh (B) enzymatic activity respectively. The mean line represents linear correlation between the gentamicin sensitivity and complexes activities A: R^2^ = 0,86593; B: R^2^ = 0,77648. Error bars represent the standard deviation of three independent experiments.
(EPS)

S1 Table Strains and plasmids used in this study. (DOCX)

S2 Table Oligonucleotides used in this study. (DOCX)
