# Supplementary material for: A small RNA controls bacterial sensitivity to gentamicin during iron starvation
Source: PLoS Genet. 2019 Apr 22;15(4):e1008078. doi: 10.1371/journal.pgen.1008078 (PMC6497325; doi:10.1371/journal.pgen.1008078)
Supplement: S1 Table — (DOCX) [file pgen.1008078.s008.docx]

| Strain /plasmid | Description | Reference |
| --- | --- | --- |
| MG1655 | *E. coli* WT | Parental strain |
| SC002 | MG1655 *ryhB ::cat* | This study |
| BEFB20 | MG1655 *nuo::npt1 ΔsdhB* | [1] |
| SC024 | BEFB20 *ryhB ::cat* | This study |
| PM1205 | MG1655 *mal*::*lacI^q^, ΔaraBAD, lacI'*:: P*_BAD_-cat-sacB:lacZ, mini  tet^R^* | [2] |
| SC005 | PM1205 *lacI'::*P*_BAD_-nuoA-lacZ* | This study |
| SC006 | SC005 *ryhB ::cat* | This study |
| SC009 | PM1205 *lacI'::*P*_BAD_-sdhC-lacZ* | This study |
| SC010 | SC009 *ryhB ::cat* | This study |
| SC026 | PM1205 *lacI'::*P*_BAD_-nuoAmut-lacZ* | This study |
| SC030 | PM1205 *lacI'::*P*_BAD_-sdhCmut-lacZ* | This study |
| SC048 | *ΔiscUA* | This study |
| SC084 | *ΔiscUA ryhB ::cat* | This study |
| BP198 | *Δsuf* | Lab collection |
| SC012 | *Δsuf ryhB ::cat* | This study |
| BEFB05 | MG1655 *nuo::npt1* Kan^R^ | [1] |
| SC085 | MG1655 *nuo::npt1 ryhB ::cat* | This study |
| BEFB06 | MG1655 *ΔsdhB* Kan^R^ | [1] |
| SC086 | MG1655 *ΔsdhB ryhB ::cat* | This study |
| plasmids |  |  |
| pBR-plac | AmpR | [3] |
| pRyhB | AmpR, Aat-II-EcoR1 *ryhB* cloned in pBR-plac | [3] |
| pRyhBmut | AmpR, Aat-II-EcoR1 *ryhB* GC5253CG cloned in pBR-plac | [4] |

Table S1. Strains and plasmids used in this study

1. Ezraty B, Vergnes A, Banzhaf M, Duverger Y, Huguenot A, Brochado AR, et al. Fe-S cluster biosynthesis controls uptake of aminoglycosides in a ROS-less death pathway. Science. 2013 Jun 28;340(6140):1583–7.

2. Mandin P, Gottesman S. A genetic approach for finding small RNAs regulators of genes of interest identifies RybC as regulating the DpiA/DpiB two-component system. Mol Microbiol. 2009;72: 551–565.

3. Mandin P. Genetic screens to identify bacterial sRNA regulators. Methods Mol Biol Clifton NJ. 2012;905:41–60.

4. Mandin P, Chareyre S, Barras F. A Regulatory Circuit Composed of a Transcription Factor, IscR, and a Regulatory RNA, RyhB, Controls Fe-S Cluster Delivery. mBio. 2016 20;7(5).
