# Supplementary material for: A small RNA controls bacterial sensitivity to gentamicin during iron starvation
Source: PLoS Genet. 2019 Apr 22;15(4):e1008078. doi: 10.1371/journal.pgen.1008078 (PMC6497325; doi:10.1371/journal.pgen.1008078)
Supplement: S2 Table — (DOCX) [file pgen.1008078.s009.docx]

Table S2. Oligonucleotides used in this study

| Name | 5’ to 3’ sequences |
| --- | --- |
| P_BAD_-nuoA-F | ACCTGACGCTTTTTATCGCAACTCTCTACTGTTTCTCCATTCTAATTATCCTGGAGTCGTCAA |
| LacZ-nuoA-R | TAACGCCAGGGTTTTCCCAGTCACGACGTTGTAAAACGACGATGACTTCAGTGGATGTTG |
| P_BAD_-sdhC-F | ACCTGACGCTTTTTATCGCAACTCTCTACTGTTTCTCCATTCTCCGGAACACCCTGCAATC |
| LacZ-sdhC-R | TAACGCCAGGGTTTTCCCAGTCACGACGTTGTAAAACGACAGGTCTTTGTTTTTTCACATTTC |
| LacI-F | TACGTTGACACCATCGAATGG |
| Deep-lac | CCGGGCCTCTTCGCTA |
| NuoA-mut-F | AAGCAATGTCAATGTCAACATCCACTGAAGTCA |
| NuoA-mut-R | GTTGACATTGACATTGCTTACTCATCAAAAGTAG |
| RyhB-mut1-F | AGCACGACATTGCTCACATTCGTTCCAGTATTACTTAGCC |
| RyhB-mut1-R | GGCTAAGTAATACTGGAACGAATGTGAGCAATGTCGTGCT |
| RyhB-mut2-F | AGAACAGCTAGTGGGCGTTATTCatgATAAG |
| RyhB-mut2-R | ACGCCCACTAGCTGTTCTTATTATTCCC |
